# Lyophilized apoptotic vesicle-encapsulated adhesive hydrogel sponge as a rapid hemostat for traumatic hemorrhage in coagulopathy

**DOI:** 10.1186/s12951-023-02128-2

**Published:** 2023-11-03

**Authors:** Yexiang Jiang, Meng Hao, Fenglin Jiang, Jiwu Li, Kunkun Yang, Can Li, Lan Ma, Shiyu Liu, Xiaoxing Kou, Songtao Shi, Xin Ding, Xiao Zhang, Jianxia Tang

**Affiliations:** 1 Hospital of Stomatology, Guanghua School of Stomatology, Sun Yat-Sen University, South China Center of Craniofacial Stem Cell Research, Guangdong Provincial Key Laboratory of Stomatology, Guangzhou, 510055 China; 2 School of Pharmaceutical Science (Shenzhen), Shenzhen Campus of Sun Yat-Sen University, Shenzhen, 518107 China; 3 Hunan Key Laboratory of Oral Health Research & Hunan Clinical Research Center of Oral Major Diseases and Oral Health, Xiangya School of Stomatology, Xiangya Stomatological Hospital, Central South University, Changsha, 410000 China; 4 Department of Prosthodontics, Peking University School and Hospital of Stomatology, National Center of Stomatology, National Clinical Research Center for Oral Diseases, National Engineering Laboratory for Digital and Material Technology of Stomatology, NHC Key Laboratory of Digital Technology of Stomatology, Beijing Key Laboratory of Digital Stomatology, Beijing, 100081 China; 5 State Key Laboratory of Military Stomatology & National Clinical Research Center for Oral Diseases & Shaanxi International Joint Research Center for Oral Diseases, Center for Tissue Engineering, School of Stomatology, The Fourth Military Medical University, Xi’an, People’s Republic of China

**Keywords:** Apoptotic vesicles, Exosomes, Hemostasis, Hydrogel sponge, Lyophilization, Hemorrhage, Coagulopathy

## Abstract

Rapid hemostasis of uncontrolled bleeding following traumatic injuries, especially accompanied by coagulopathies, remains a significant clinical challenge. Extracellular vesicles (EVs) show therapeutic effects for fast clotting. However, low yield, specific storage conditions, and lack of proper carriers have hindered EVs’ clinical application. Herein, we establish an optimized procedure method to generate lyophilized mesenchymal stem cell-derived apoptotic vesicles (apoVs) with adhesive hydrogel sponge to show superior procoagulant activity for traumatic hemorrhage. Mechanistically, apoVs’ procoagulant ability stems from their high tissue factor (TF) and phosphatidylserine (PS) expression independent of hemocytes and circulating procoagulant microparticles (cMPs). Their stable hemostatic capability was maintained after 2-month room temperature storage. Subsequently, we mixed apoVs with both phenylboronic acid grafted oxidized hyaluronic acid (PBA-HA) and poly(vinyl alcohol) (PVA) simultaneously, followed by lyophilization to construct a novel apoV-encapsulated hydrogel sponge (apoV-HS). Compared to commercial hemostats, apoV-HS exhibits rapid procoagulant ability in liver-laceration and femoral artery hemorrhage in rat and rabbit models of coagulopathies. The combination of high productivity, physiological stability, injectability, plasticity, excellent adhesivity, biocompatibility, and rapid coagulant property indicates that apoV-HS is a promising therapeutic approach for heavy hemorrhage in civilian and military populations.

**Graphical Abstract:**

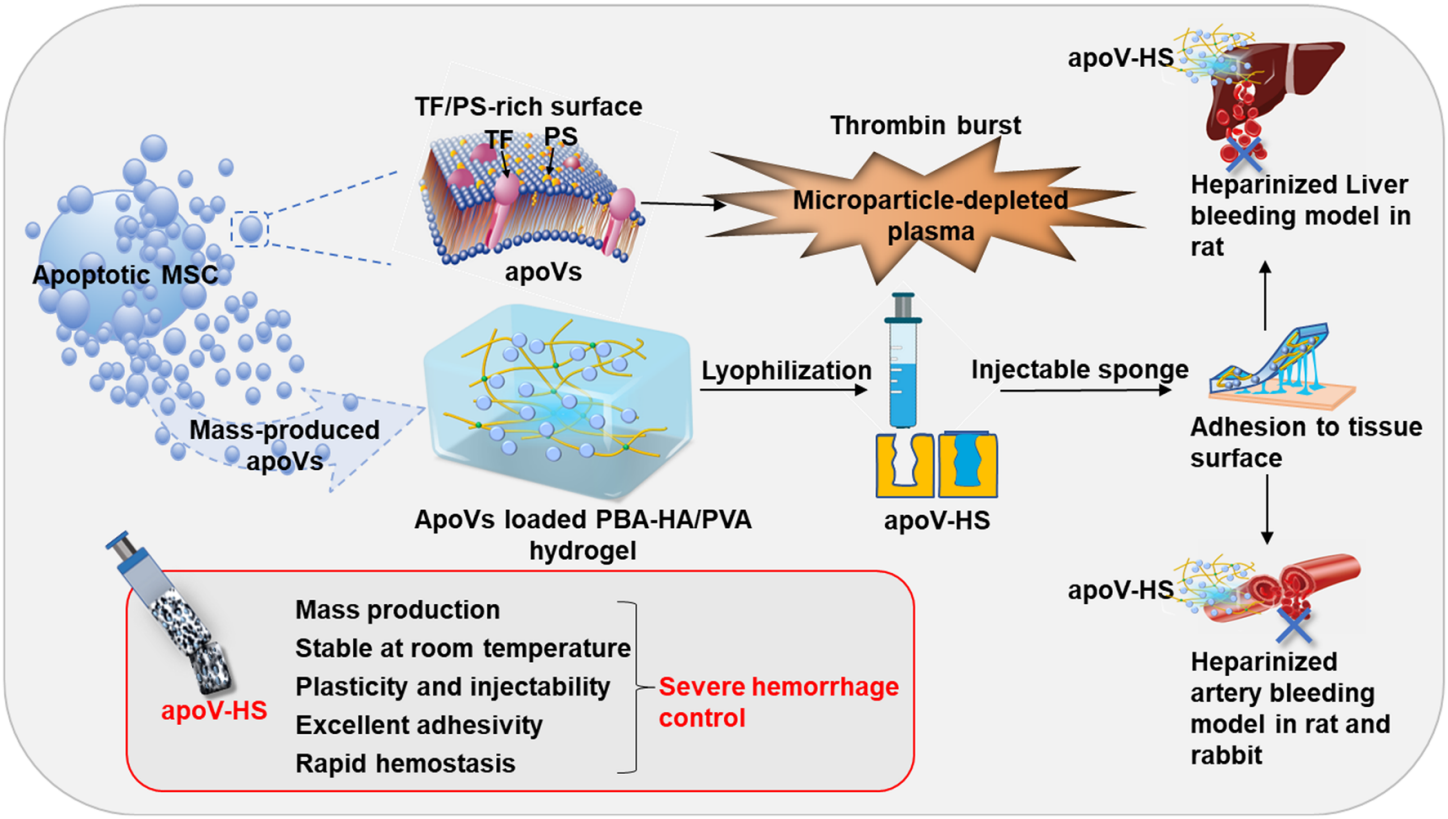

**Supplementary Information:**

The online version contains supplementary material available at 10.1186/s12951-023-02128-2.

## Introduction

Uncontrolled traumatic bleeding leads to over 80% of battlefield mortality [1] and more than 30% of civilian traumatic deaths [2, 3]. In addition, severe traumatic wounds can often result in coagulopathies, where the blood clotting ability of the body is impaired [4, 5]. As a consequence, compared to trauma patients without coagulopathies, those with coagulopathies have a four- to six-fold greater mortality [6]. To date, various topical hemostatic agents are applied in the clinic [7, 8]. However, fast and effective hemostatic control of life-threatening bleeding, especially associated with coagulopathies remains a significant clinical challenge. Therefore, developing a novel portable hemostat to stop severe bleeding quickly, especially on the battlefield, is highly desirable.

Accumulating evidence indicates that circulating microparticles (cMPs) could promote coagulation via their exposure of tissue factor (TF) and phosphatidylserine (PS) [9, 10], and reduce significantly in trauma patients under coagulopathic conditions [11, 12]. However, owing to their donor dependence, high cost with low yield, and potential contamination, cMPs are difficult to be applied clinically to stop bleeding. Hence, it is worth producing TF and PS containing-procoagulant extracellular vesicles (EVs) derived from cells in mass production in vitro. EVs can be divided into three main categories: exosomes (Exos), microvesicles (MVs), and apoptotic vesicles (apoVs), all of which have been proven to express varying degrees of TF and PS [13–15]. Compared with Exos and MVs, apoVs are more suitable for clinical application because of their higher yield, shorter preparation time, lower cost, etc. Intriguingly, we also found that MSC-derived apoVs could ameliorate haemophilia A via activating platelet functions, which implies their potential use in the hemostatic field [16]. However, whether topical application of MSC-derived apoVs can quickly stop heavy traumatic bleeding is still unclear.

Severe traumatic hemorrhage accompanied by coagulopathies is life-threatening and requires fast hemostatic control, which pushes us to consider how to keep both EVs and their carrier with high portability, stability, and practicability when we aim to enable them to be applied as a topical hemostat. Lyophilized EVs as dry powder can be stored at room temperature (RT) for the long term, which resolves the issues of storage, bioactivity, and transport [17–19]. However, it is not practical for the powder to stop heavy bleeding because it is soluble in blood and thus cannot be fixed on the wound surface. As hydrogels are porous and highly compatible with biomacromolecules, cells, and tissue [20, 21], they have been widely used as ideal carriers to load EVs and maintain their bioactivity for the treatment of skin wounds, bone defects and periodontitis [22–24]. Moreover, hydrogel after lyophilization, as hydrogel sponge, can be stored under dry conditions at RT [25, 26], which may make lyophilized EV-loaded hydrogel portable and long-preserved. In addition, the hydrogels alone had been applied as topical hemostatic materials owing to their tissue adhesive property after in situ gelation [7, 27]. However, such hemostatic hydrogels have to be prepared on site and need irradiation of ultraviolet light or addition of strong oxidant solution such as NalO_4_ for gelation, which restricts their application for rapid control of heavy hemorrhage [27–29]. The hydrogels based on phenylboronic acid grafted hyaluronic acid and poly(vinyl alcohol) (PVA) have been studied for tissue regeneration [30], wound healing [31], and cell delivery [32], as both hyaluronic acid (HA) and PVA are medically used biocompatible macromolecules, and dynamic boronic ester bond formed between boronic acid and diol groups of PVA endows the hydrogel with injectability and plasticity. Meanwhile, the partial oxidization of HA to generate aldehydes could facilitate adhesive fixation of hydrogel to tissue, which is critical for hemostasis [33]. Therefore, a pre-prepared EV-encapsulated hydrogel sponge, which is composed of phenylboronic acid grafted oxidized HA (PBA-HA) and PVA, with strong tissue adhesivity, injectability, and plasticity, could be a clinical hemostat. To the best of our knowledge, there is no study of EV-encapsulated hydrogel sponge that has been explored for hemostasis.

Herein, we first established an optimized procedure to produce umbilical cord mesenchymal stem cell-derived apoVs with high yield, and confirmed their faster clotting property in vitro compared to Exos. We further demonstrated that apoVs could promote plasma clotting in the absence of hemocytes and cMPs through the extrinsic pathway in vitro, which implies apoVs’ potential application for stopping coagulopathic bleeding. In addition, we lyophilized apoVs and verify their stable hemostatic capability even after 2-month RT storage in vitro. Moreover, we constructed a novel portable apoV-encapsulated adhesive hydrogel sponge (apoV-HS) by lyophilization of apoVs with PBA-HA and PVA. Subsequently, we verified apoV-HS’ excellent ability to accelerate hemostasis in traumatic bleeding animal models with coagulopathies.

## Results and discussion

### Human umbilical cord mesenchymal stem cell (UCMSC)-derived apoptotic vesicles (apoVs) highly express tissue factor (TF) and phosphatidylserine (PS)

**Fig. 1:**
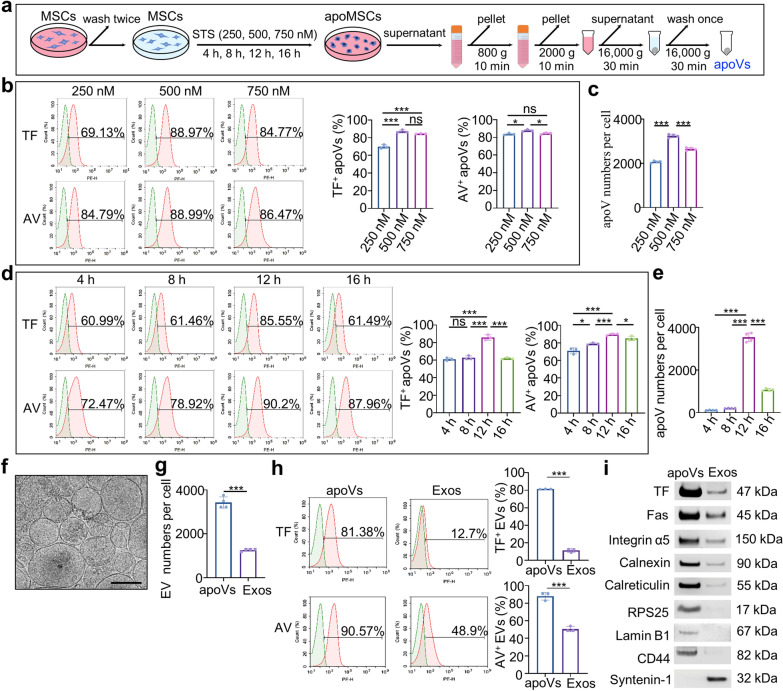
UCMSC-derived apoptotic vesicles (apoVs) highly express tissue factor (TF) and phosphatidylserine (PS). **a** Schematic diagram showing the procedures of isolating apoVs. STS, staurosporine; apoMSCs, apoptotic MSCs. **b** Flow cytometric analysis and the quantification of the percentages of TF^+^ or Annexin V (AV)^+^ apoVs. Surface exposure of PS was confirmed by the percentages of AV binding rate. ApoVs were collected from UCMSCs treated with different concentrations of STS (250, 500, and 750 nM) for 12 h. n = 3 per group. **c** Nanoparticle tracking analysis (NTA) and the quantification of apoVs’ yield. n = 4 per group. **d** Flow cytometric analysis and the quantification of the percentages of TF^+^ or AV^+^ apoVs. ApoVs were collected from UCMSCs treated with 500 nM STS for 4, 8, 12, and 16 h, respectively. n = 3 per group. **e** NTA and the quantification of apoVs’ yield. n = 4 per group. **f** Cryo-electron microscopy (Cryo-EM) showing the morphology of apoVs. Scale bar, 200 nm. **g** NTA and the quantification of yield of apoVs and exosomes (Exos) derived from the same number of UCMSCs. n = 4 per group. **h** Flow cytometric analysis and the quantification of the percentages of TF^+^ or AV^+^ extracellular vesicles (EVs). n = 3 per group. **i** Western blotting analysis showing the inclusive (8 proteins) and exclusive (Syntenin-1) biomarkers of apoVs. ns, no significant; *p < 0.05; ***p < 0.001

Human UCMSCs were used in this study due to their non-invasive harvest procedure and fast self-renewal properties in vitro [34]. We verified that UCMSCs expressed MSC markers (CD29, CD44, CD90), but not hematopoietic markers (CD34 and CD45) by flow cytometric analysis (Additional file 1: Figure S1). UCMSC-derived apoptotic vesicles (apoVs) were prepared by gradient centrifugation as described in our previous study (Fig. 1a) [16]. Numerous studies have demonstrated that EVs promote coagulation mainly via their surface exposure of TF and PS [9, 10, 35]. Hence, to acquire procoagulant apoVs for rapid hemostasis, we used flow cytometry analysis to detect the expression of TF and PS in apoVs obtained from UCMSCs. To optimize the procedure, in this study, we used different concentrations of STS at different times to induce UCMSC apoptosis to acquire the optimal apoVs. The results showed that the expressions of both TF and PS were highest in apoVs when they were collected from UCMSCs induced by 500 nM STS (Fig. 1b). Next, UCMSCs were treated with 500 nM STS for 4, 8, 12, and 16 h, respectively, and the data confirmed that apoVs collected from UCMSCs induced by 500 nM STS for 12 h had the highest expression of both TF and PS (Fig. 1d). Meanwhile, nanoparticle tracking analysis (NTA) by Zetaview demonstrated that the yield of apoVs was also highest when they were obtained from UCMSCs induced by 500 nM STS for 12 h (Fig. 1c, e). Therefore, we chose apoVs obtained from UCMSCs induced by 500 nM STS for 12 h for our following studies.

Next, apoVs were characterized by Cryo-electron microscopy (Cryo-EM), NTA, flow cytometry, and western blotting. Cryo-EM showed that apoVs had an intact membrane spherical structure (Fig. 1f). NTA determined that the diameter of apoVs ranged from 10 to 1000 nm, the median diameter was about 198.2 nm (Additional file 1: Figure S2a), and the mean membrane potential was approximately − 37.85 mV (Additional file 1: Figure S2b). The flow cytometric analysis demonstrated that apoVs highly expressed apoptotic imprint Fas, EV biomarkers (CD9, CD63, CD81), and MSC markers (CD29, CD44, CD90), but not hematopoietic markers (CD34 and CD45) (Additional file 1: Figure S2c). Next, we used NTA to find that about 3400 apoVs were derived from only one cell and the yield of apoVs was significantly higher than that of Exos nearly threefold (Fig. 1g), which laid the foundation for the future clinical application of apoVs. More importantly, the flow cytometric analysis showed that apoVs expressed a significantly higher level of TF and PS compared to Exos (Fig. 1h). Western blotting results further confirmed that apoVs had a higher expression of TF compared to exos (Fig. 1i). Consistent with our previous study [16], Fas, Integrin α5, calnexin, calreticulin, RPS25, Lamin B1, and CD44 were specific biomarkers, while syntenin-1 was an exclusive biomarker for apoVs (Fig. 1i). These data together illustrate that TF and PS are highly expressed in apoVs. Previous studies have determined that TF^+^ & PS^+^ EVs exhibit the best procoagulant efficiency compared with TF^+^ EVs or PS^+^ EVs [14, 36]. Therefore, it seems that apoVs may quickly initiate coagulation cascades and thus have the potential to further act as an efficient hemostat.

### ApoVs’ procoagulant property is independent of hemocytes and cMPs and stems from their highly expressed TF and PS

**Fig. 2:**
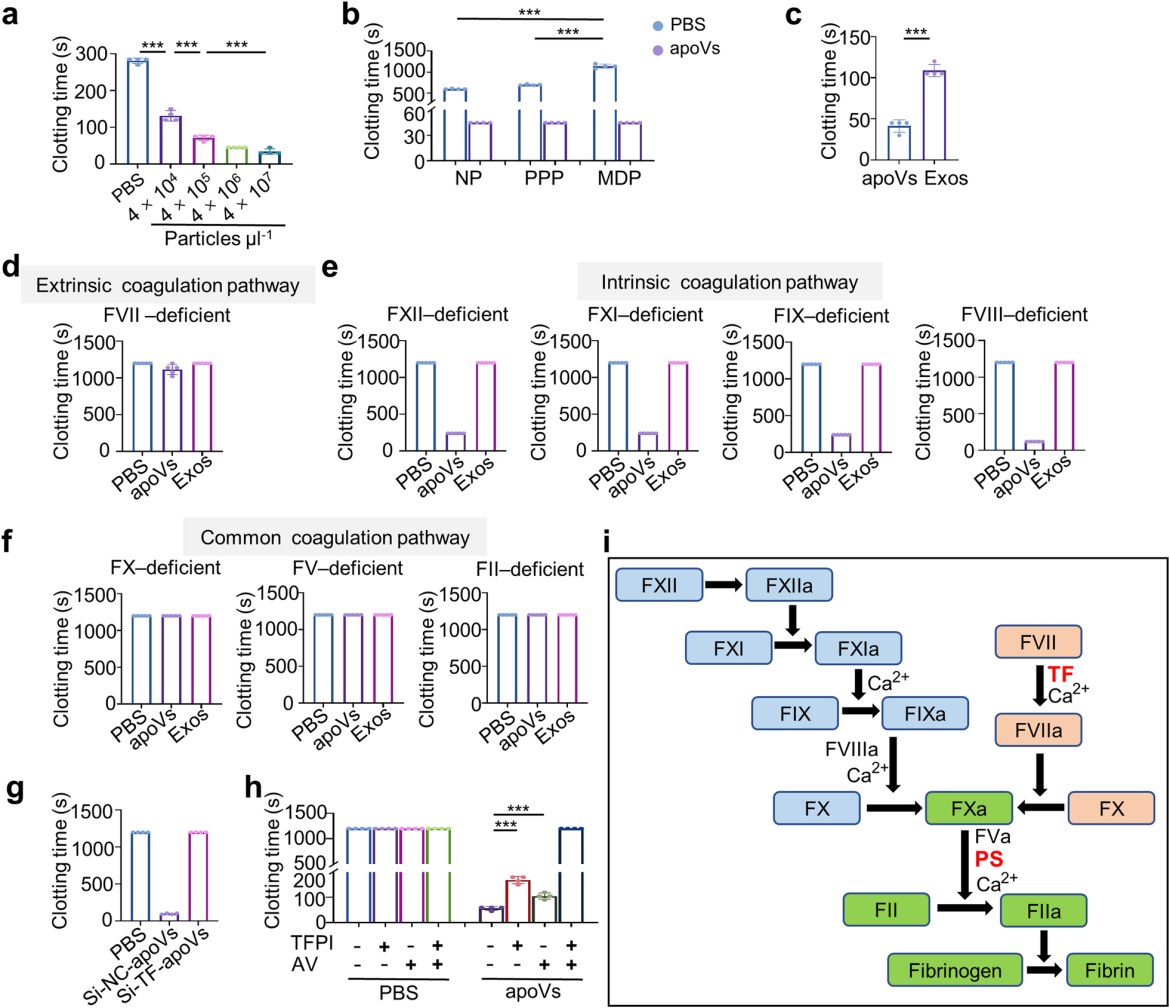
ApoVs promote plasma clotting independent of hemocytes and microparticles and stemmed from TF and PS*.* **a** The clotting time of rat whole blood treated with different concentrations of apoVs. n = 4 per group. **b** The clotting time of rat normal plasma (NP), platelet-poor plasma (PPP), and microparticle-depleted plasma (MDP) treated with apoVs. n = 4 per group. **c** The clotting time of rat MDP treated with the same concentration of apoVs or Exos. n = 4 per group. **d**–**f** The clotting time of human coagulation factor-deficient plasma treated with apoVs or Exos. n = 5 per group. **g** The clotting time of human MDP treated with si-NC-apoVs or si-TF-apoVs. n = 4 per group. si-NC-apoVs, apoVs derived from UCMSCs treated with siRNA-negative control; si-TF-apoVs, apoVs derived from UCMSCs treated with siRNA-TF. **h** The clotting time of human MDP with indicated treatment. Tissue factor pathway inhibitor (TFPI) was used to block TF function and AV was used to block PS function. n = 4 per group. **i)** Diagram illustrating multi-step pathways including intrinsic (blue), extrinsic (orange), common (green) coagulation pathways, and the role of TF and PS (red) from apoVs to promote coagulation. The clotting time of plasma that exceeded 1200 s was recorded as “1200”. ***p < 0.001

EVs are known to enhance coagulation and thus participate in hemostasis [37]. However, current studies mainly showed the hemostatic efficiency and potential application of EVs in mild bleeding conditions [38, 39]. Whether EVs could be utilized in severe hemorrhage associated with coagulopathies is largely unknown. To test whether apoVs are superior EV candidates for fast control of severe hemorrhage, we first conducted clotting experiments in vitro. The results revealed that apoVs can dose-dependently shorten the clotting time of rat whole blood (Fig. 2a). Considering that there is no significant difference in clotting time between treatments with the dose of apoVs at 4 × 10^6^ particles μl^−1^ (45 s) and 4 × 10^7^ particles μl^−1^ (33.75 s), we chose the dose 4 × 10^6^ particles μl^−1^ for further experiments. Considering the importance of circulating microparticles (cMPs) in blood coagulation [40] and the similarity between cMPs and apoVs, we speculate that apoVs may directly replace the procoagulant function of cMPs and accelerate hemostasis. Hence, we compared the procoagulant effect of apoVs on rat normal plasma (NP), platelet-poor plasma (PPP), and microparticle-depleted plasma (MDP), in which 90% of cMPs were removed. The results showed that the clotting time of MDP was significantly prolonged compared to that of NP and PPP in PBS groups, while apoV treatment all significantly decreased the clotting time in these 3 kinds of plasma (Fig. 2b). More interestingly, there was no difference in the clotting time of rat NP, PPP, and MDP with apoV treatment (Fig. 2b). These results together demonstrated that cMPs alone indeed play vital roles in fast clotting and apoVs could replace them to promote coagulation independent of hemocytes and cMPs. Moreover, apoVs significantly decreased the clotting time of rat MDP compared to the same concentration of Exos treatment (Fig. 2c), indicating the superior procoagulant performance of apoVs.

To further elucidate how apoVs play the procoagulant function in the coagulation cascade, we used a series of human coagulation factor-deficient plasma to explore which kinds of coagulation factors are involved in apoV-mediated fast clotting. Notably, the clotting time of plasma that exceeded 1200 s was recorded as “1200”. The results showed that both apoVs and Exos could not reduce the clotting time of factor VII (extrinsic coagulation factor), X, V, and II (common coagulation factors)-deficient plasma (Fig. 2d, f). Meanwhile, Exos but not apoVs could not decrease the clotting time of factor XII, XI, IX, and VIII-deficient plasma (Fig. 2e), which were intrinsic coagulation factor-deficient plasma. These data together indicate that apoVs’ procoagulant ability is mainly through the extrinsic coagulation pathway while that of Exos is dependent on both extrinsic and intrinsic coagulation pathways.

It is widely accepted that TF: fVIIa complex is an extremely potent activator in the coagulation cascade [41, 42]. Although soluble PS can trigger the formation of a soluble prothrombinase complex, the phospholipid anchoring region of TF is necessary for autoactivation and beneficial for factor Xa-catalyzed activation of fVII [43, 44]. The TF: fVIIa complex formation can be damaged due to the lack of membrane structure [45, 46]. Thus, the form of membrane-carried TF is necessary for effective hemostasis. To explore whether TF in apoVs plays a role in apoV-mediated fast clotting, we used siRNA technology to knock down TF expression in UCMSCs and then collected UCMSC-derived apoVs. Reduced expression of TF in UCMSCs and UCMSC-derived apoVs were verified by western blotting (Additional file 1: Figure S3a, b). The clotting experiment showed that apoVs with TF knockdown (Si-TF-apoVs) failed to accelerate the clotting of human MDP (Fig. 2g). Considering that PS could also accelerate thrombin generation by bringing different components of coagulation cascades together [47], to further explore whether PS from apoVs also contributes to coagulation, we blocked surface TF or PS of apoVs with tissue factor pathway inhibitor (TFPI), a fXa-dependent inhibitor of TF-fVIIa [48], or Annexin V (AV) as described previously [49], and then performed human MDP clotting experiment. The results showed that the procoagulant effects of apoVs on plasma clotting were impaired by TF-fVIIa or PS blockade (Fig. 2h). Additionally, the combined pretreatment of TFPI and AV completely inhibited the procoagulant ability of apoVs, generating a synergic effect (Fig. 2h), which was consistent with a previous study [14]. Collectively, these results suggest that apoVs significantly promote plasma clotting by initiating the extrinsic coagulation pathway via TF and PS in vitro (Fig. 2i).

### The bioactivity and procoagulant ability of apoVs are preserved by lyophilization

**Fig. 3:**
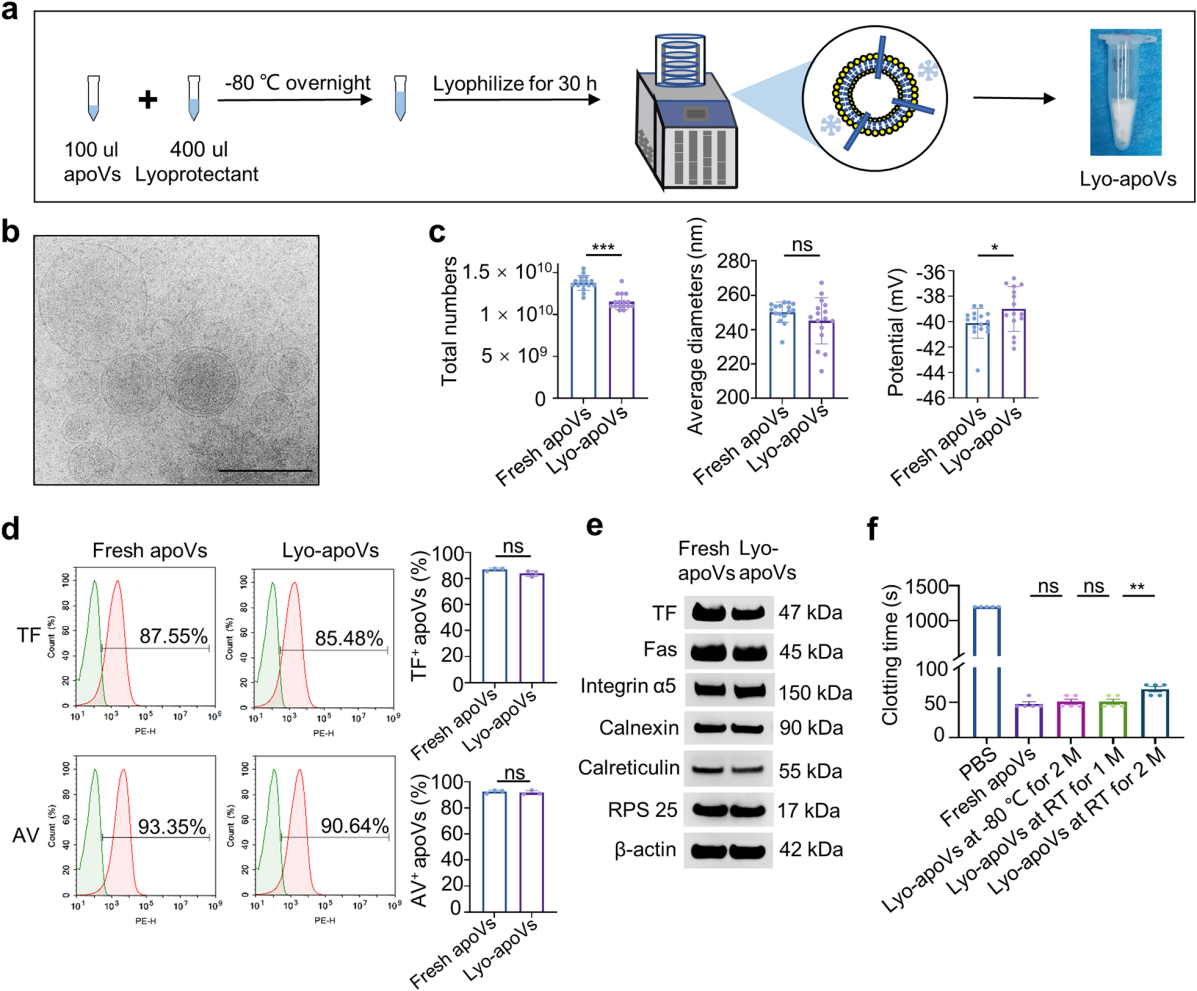
The bioactivity and procoagulant ability of apoVs are preserved by lyophilization. **a** Schematic diagram indicating the lyophilization procedures of apoVs. Lyo-apoVs, lyophilized apoVs. **b** Cryo-EM showing the morphology of lyo-apoVs. Scale bar, 200 nm. **c** NTA and the quantification of total numbers, average diameters, and membrane potentials of apoVs. n = 16 per group. **d** Flow cytometric analysis and the quantification of the percentages of TF^+^ or AV^+^ apoVs. n = 3 per group. **e** Western blotting showing the expression levels of TF and inclusive apoVs biomarkers (5 proteins) in fresh and lyophilized apoVs. **f** The clotting time of rat MDP treated with fresh apoVs or lyo-apoVs from different storage conditions (− 80 °C for 2 months, RT for 1 month, and RT for 2 months). n = 5 per group. ns, no significant; *p < 0.05; **p < 0.01; ***p < 0.001

So far, plenty of topical hemostatic agents are available for effective control of normal bleeding in the clinic. However, there is no optimal product used for fast control of severe traumatic hemorrhage in coagulopathies. Therefore, it is very desirable to explore the potential of EV-based hemostats in stopping heavy bleeding under the coagulopathic state. Lyophilization represents an ideal approach for EV-based therapy, because it resolves long-term storage challenges, guarantees bioactivity, prevents cross-contamination, and facilitates the transport of EV products [18, 19]. Thus, to facilitate the storage and carriage of apoVs, we established optimized lyophilization technologies to acquire freeze-dried apoVs (Fig. 3a) as previously reported [17]. Lyophilized apoVs were characterized by Cryo-EM, NTA, flow cytometry, and western blotting. Cryo-EM images showed that lyophilized apoVs were intact membrane spherical structures (Fig. 3b), indicating that the integrity of apoVs was preserved after lyophilization. Next, NTA was used to compare the total numbers, average diameters, and membrane potentials between fresh and lyophilized apoVs. The results revealed that the diameters of apoVs did not change after lyophilization, while the total numbers and membrane potentials of lyophilized apoVs were changed compared to the fresh apoVs (Fig. 3c). In addition, flow cytometry analysis revealed that there was no significant difference in surface exposure of TF and PS between fresh and lyophilized apoVs (Fig. 3d). Moreover, western blotting showed that the expression of TF in lyophilized apoVs was only slightly decreased, while there was no significant difference in the expression of apoV biomarkers between lyophilized apoVs and fresh apoVs (Fig. 3e). These results suggest that most of the apoVs’ properties are kept by freeze-drying technology. Next, to confirm the hemostatic function of lyophilized apoVs, we compared the procoagulant ability of fresh apoVs and lyophilized apoVs from different storage conditions. Importantly, fresh apoVs, lyophilized apoVs after − 80 °C of storage for 2 months, or RT storage for 1 month possessed similar procoagulant effect on rat MDP (Fig. 3f). Although the lyophilized apoVs after RT storage for 2 months showed slightly inferior procoagulant effect than three other apoVs, they still significantly decreased clotting time than PBS group (Fig. 3f). The slight change of membrane potentials or relevant protein expression showed less effect on the procoagulant ability of lyophilized apoVs. Overall, these results together indicate that the bioactivity of lyophilized apoVs are well preserved, and the procoagulant properties of lyophilized apoVs are even stable at RT storage for at least 2 months in vitro.

### Preparation and characterization of apoV-encapsulated hydrogel sponge (apoV-HS)

**Fig. 4:**
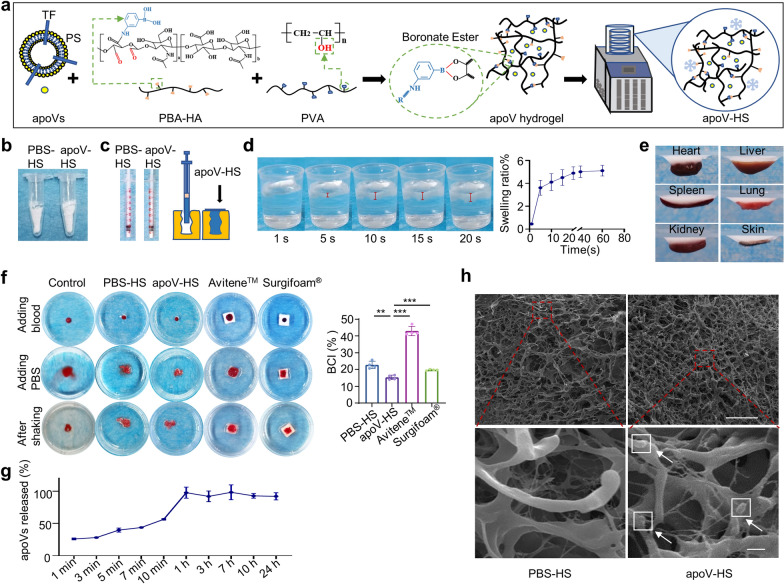
Preparation and characterization of apoV-encapsulated hydrogel sponge (apoV-HS). **a** Schematic diagram indicating the assembly of apoV-HS and the procedures of lyophilization. PBA-HA, phenylboronic acid grafted oxidized hyaluronic acid. PVA, poly(vinyl alcohol). Yellow circles with blue border represent apoVs in the diagram. **b** Digital photos showing the morphology of lyophilized hydrogel sponge. PBS-HS, hydrogel sponge with PBS added during assembly. **c** Digital photos and diagram showing the compression property and injectability of PBS-HS and apoV-HS. **d** Digital images and statistical analysis illustrating the swelling processes of apoV-HS in deionized water (DI). n = 3 per group. **e** Digital photos demonstrating the adhesive property of apoV-HS to the surface of mice organs. **f** Digital photos illustrating process of blood clotting upon hemostatic agents and quantification of the blood clotting index (BCI). n = 4 per group. **g** Quantification of apoVs’ release efficiency from apoV-HS. n = 4 per group. **h** Scanning electron microscope (SEM) showing the morphology of lyophilized hydrogel sponge after hydration. The white arrows indicate apoVs. Scale bar, 25 μm (upper panel); 1 μm (lower panel). **p < 0.01; ***p < 0.001

Inspired by previous reports [26, 50], we innovatively fabricated apoV-encapsulated hydrogel sponge (apoV-HS) through cross-linking of phenylboronic acid grafted oxidized hyaluronic acid (PBA-HA) with poly(vinyl alcohol) (PVA), simultaneous encapsulation of apoVs, and subsequent freeze-drying procedure (Fig. 4a, b). Although the lyophilization technique has been utilized for PBA-HA or PVA material, respectively [32, 51, 52], whether the composite of PBA-HA and PVA could be lyophilized is unknown. Therefore, our study is the first attempt to lyophilize PBA-HA and PVA together to test whether this manner could generate a novel injectable hydrogel sponge. Notably, this apoV-HS is compressible, plastic, and injectable probably due to the dynamic boronic ester bond (Fig. 4c), indicating potential applications in deep, irregular, and difficult-to-access trauma cavities. Existing topical hemostats mostly is not efficient in controlling hemorrhage from arterial and cardiac wounds because of their weak adhesion to wet tissues [28]. Our results show that once encountering water, the compressed apoV-HS rapidly expanded within 20 s, absorbed in water, and turned into an adhesive hydrogel, which could adhere tightly to the surface of tissues and organs (Fig. 4d, e). The strong adhesive property could be attributed to the aldehyde and phenylboronic acid groups in PBA-HA which can form chemical bonds with biomacromolecules in tissues containing amine and polyhydroxy groups, respectively [33].

We next compared the apoV-HS to commercial hemostatic sponge Avitene™ (collagen sponge) and Surgifoam® (gelatin sponge) in their blood clotting index (BCI). The digital photos showed that in the blood clotting index process, the suspension in apoV-HS, PBS-HS, and Surgifoam® groups remained clear even after shaking, while the suspension in the control and Avitene™ groups were light red after shaking. A lower absorbance value means more blood cells being fixed in the blood clot, which represents the higher procoagulant capability of hemostatic materials [26]. The BCI value of apoV-HS was significantly lower than those of PBS-HS, Avitene™, and Surgifoam®, indicating a better hemostatic ability of apoV-HS in vitro (Fig. 4f). In addition, to examine the releasing rate of apoVs from apoV-HS, we immersed PKH26 labeled-apoV-HS into PBS and detected the absorbance at 570 nm at set time points. The results showed that apoV-HS had a burst release of apoVs (nearly 25%) within 1 min, and apoVs were almost totally released within 1 h (Fig. 4g). The releasing profile of encapsulated substances in hydrogel is dependent on the pore size, and the fast apoV release is probably due to the relatively large pore size of the hydrogel sponge (Fig. 4h). The releasing rate of apoV-HS was faster than other vesicle-loaded hydrogels [22, 53]. The fast-releasing profile indicates that apoVs could be quickly released to stop local bleeding and continue to be released within 1 h to prevent secondary hemorrhage. Furthermore, scanning electron microscope (SEM) images showed that apoVs were visible on the surface of hydrated apoV-HS (Fig. 4h, white arrows), insuring the direct contact of apoVs to blood, and the fastest participation into hemostasis. Previous studies showed that hydrogel loaded with extracellular vesicles have great application potential in hemostasis and regeneration [29, 54]. Therefore, we chose to load hydrogel with apoVs to improve hemostatic function. Our study, for the first time, combines apoVs, hydrogel sponge, and the lyophilization technique to construct a novel hemostatic apoV-HS. Considering the injectability and plasticity, excellent adhesivity, and rapid hemostatic property of apoV-HS in vitro, apoV-HS has the potential application for rapid severe hemorrhage control.

### ApoV-HS possesses superior biocompatibility both in vitro and in vivo

**Fig. 5:**
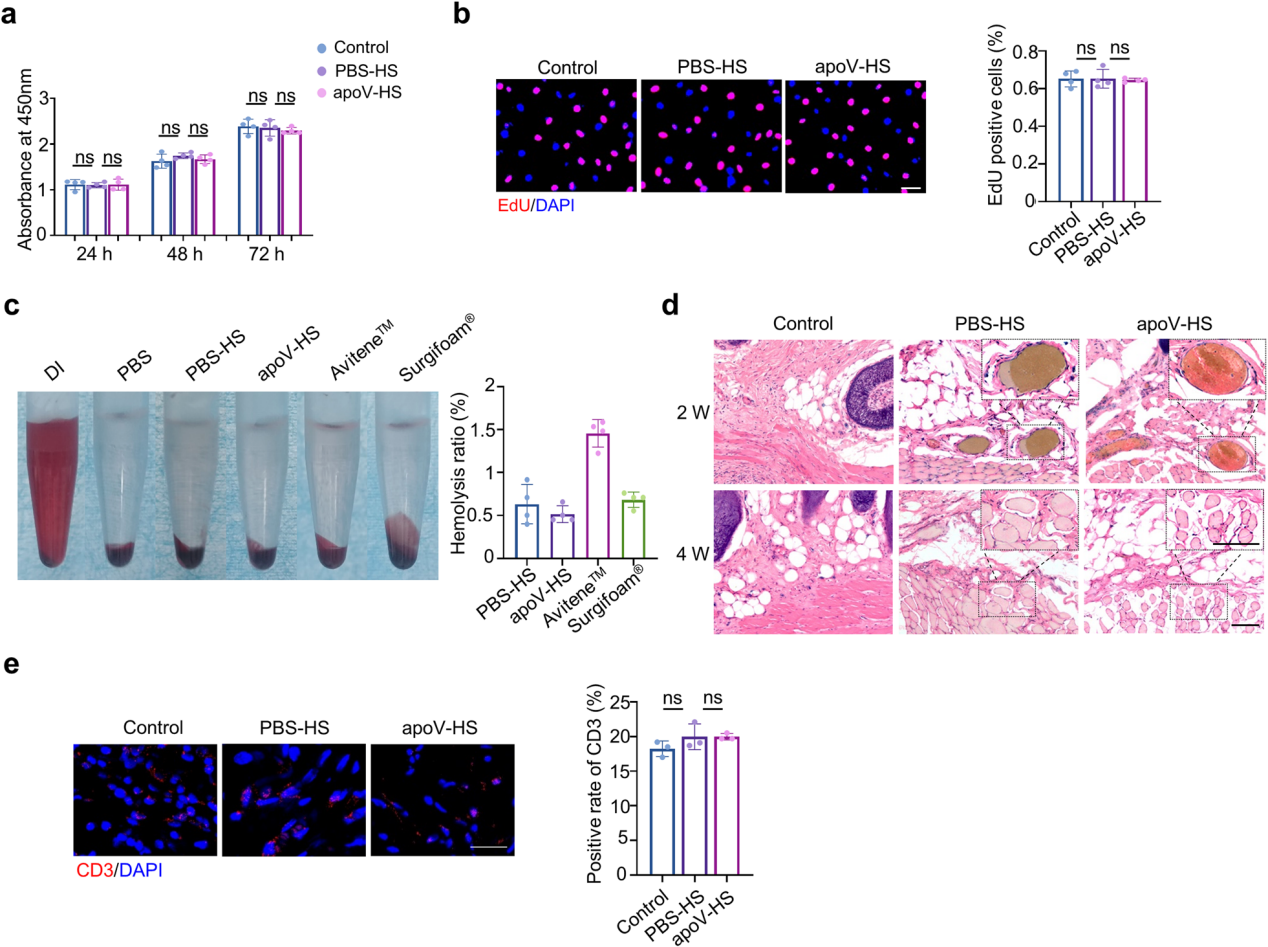
ApoV-HS possesses a superior biocompatibility both in vitro and in vivo. **a** CCK8 assay indicating the proliferation rate of L929 cells (mouse fibroblasts) treated with the leaching solutions from indicated groups. **b** Fluorescence images and quantification showing the proliferation rate of L929 cells treated with the leaching solutions from indicated groups. Scale bar, 40 μm. Red: Edu staining; Blue: DAPI staining. **c** Hemolysis rate of apoV-HS and control groups (PBS-HS, Avitene™, Surgifoam®). DI and PBS were set as positive and negative control, respectively. **d** HE staining showing histological changes of mice skin after subcutaneous implantation with PBS-HS or apoV-HS for 2 and 4 weeks. Mice in control group underwent the same surgical procedures, with no materials implanted. Scale bar, 100 μm. n = 4 per group. **e** Fluorescence images and quantification showing the CD3^+^ T cells of mice skin after subcutaneous implantation for 4 weeks. Scale bar, 20 μm. Red: CD3 staining; Blue: DAPI staining. ns, no significant

To evaluate the biocompatibility of apoV-HS, we performed systematic assays in vitro and in vivo. Both the CCK8 assay (Fig. 5a) and EdU assay (Fig. 5b) showed that the proliferation rate of L929 cells (mouse fibroblasts) was not influenced by the culture media conditioned with the leaching solutions of apoV-HS, indicating the non-cytotoxic property of apoV-HS. In addition, the hemocompatibility of apoV-HS was evaluated by hemolysis tests in vitro. The hemolysis rate of apoV-HS was 0.51% ± 0.1% (Fig. 5c), far less than the lowest international standard (5%) for the clinical use of agents [55]. Furthermore, we evaluated in vivo biocompatibility of apoV-HS on the dorsal subcutaneous implantation in rat models. Histological images showed that apoV-HS elicited very mild inflammation at 2 weeks and 4 weeks post-implantation (Fig. 5d). Immunofluorescence (IF) staining also showed that the infiltrated CD3^+^ T cells in apoV-HS group had no significant difference from control group (Fig. 5e). These results together suggest that apoV-HS has superior biocompatibility in vitro and in vivo, indicating that apoV-HS is a safe hemostatic material.

### ApoV-HS exhibits rapid hemostatic effect on heparinized rat liver bleeding and femoral artery/vein bleeding models

**Fig. 6:**
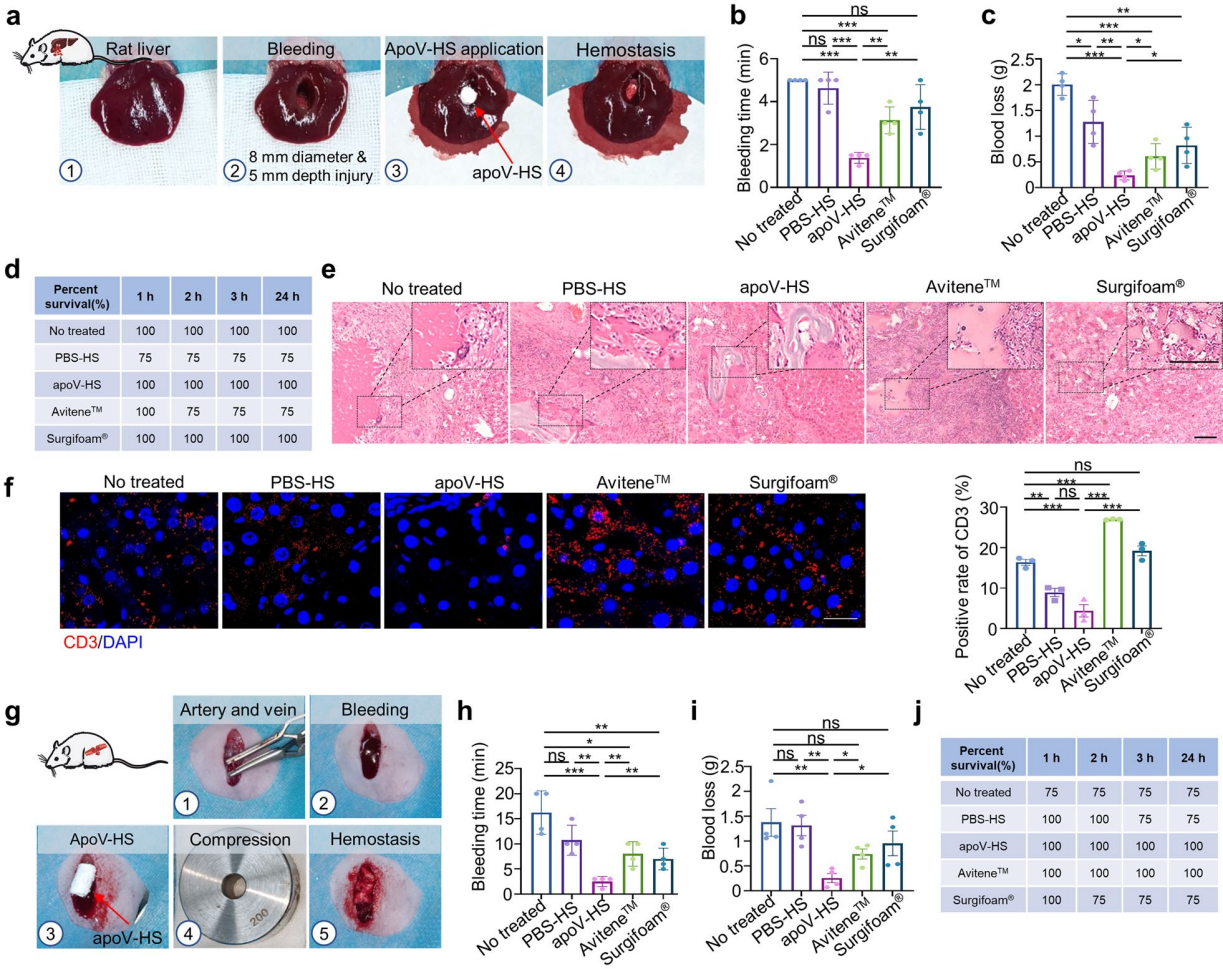
ApoV-HS exhibits rapid hemostatic effect on heparinized rat liver bleeding and femoral artery/vein bleeding models. **a** Digital photos showing hemostatic procedures for rat liver bleeding model treated with apoV-HS. **b**, **c** Bleeding time and blood loss of rat liver bleeding models with indicated treatments. The maximal bleeding time was set at 5 min. **d** The survival rate of rats with liver bleeding. **e**, **f** Representative histology and IF images of injured livers at 4 weeks post-operation. Scale bar, 100 μm (HE); Scale bar, 30 μm (IF); Red: CD3 staining; Blue: DAPI staining. **g** Digital photos showing hemostatic procedures for rat femoral artery/vein bleeding model treated with apoV-HS. **h**, **i** Bleeding time and blood loss of rat femoral artery/vein bleeding models with indicated treatments. **j** The survival rate of rats with femoral artery/vein bleeding. n = 4 per group. ns, no significant; *p < 0.05; **p < 0.01; ***p < 0.001

Although some studies have shown the application of injectable products based on PBA-HA in hemostasis [52, 56], whether this biomaterial could be used in fast control of severe bleeding remains largely unknown. Thus far, we have demonstrated the excellent hemostatic capacity of apoV-HS in vitro. Next, we used rat heparinized liver and femoral artery/vein bleeding models as simulating clinical uncontrolled hemorrhage in coagulopathies to verify the rapid procoagulant property of apoV-HS in vivo. In the rat liver bleeding model, the left anterior lobe of the liver was punctured to create a cylindrical defect (8 mm in diameter, nearly 5 mm in depth) at 30 min post-heparin injection, with simultaneous treatment with hemostatic agents (Fig. 6a). Two sponge products (Avitene™ and Surgifoam®) were chosen as representative commercial hemostats based on their good hemostatic properties. The bleeding was quickly stopped at 1.375 ± 0.25 min in the apoV-HS group, requiring significantly less time and blood loss to achieve hemostasis compared to other groups (Fig. 6b, c). ApoV-HS could quickly and effectively stop acute bleeding, so rats in the apoV-HS group did not undergo secondary bleeding and survived. 25% of rats died in the PBS-HS and Avitene™ group, respectively, due to ineffective hemostasis and secondary hemorrhage. There was no statistical difference in terms of mortality among these groups (Fig. 6d). These results indicate that apoV-HS has superior hemostatic performance in vivo. To evaluate the biocompatibility of apoV-HS, we next collected the injured rat livers at 4 weeks after surgery for histological analysis. Hematoxylin–eosin (HE) staining images showed that apoV-HS and other hemostatic agents could not be fully degraded in the injured livers at 4 weeks after surgery (Fig. 6e), indicating a longer time is needed for complete degradation of materials in livers. Notably, in the liver of the no treated group, a large area of necrotic hepatic tissue was observed, probably because of the most blood loss of the group without any treatment. In addition, apoV-HS group exhibited the least inflammatory cell infiltration, indicating good biocompatibility of apoV-HS in vivo (Fig. 6e, f). In contrast, there was a large amount of inflammatory cell infiltration in sites of injured livers by Avitene™ and Surgifoam® treatment. Although Avitene™ and Surgifoam® have already been approved to use in the clinic, there are several reports show that these materials induce obvious granulomatous inflammation or eosinophil infiltration in different tissues [57–59]. Therefore, the excellent biocompatibility of apoV-HS is very attractive for clinical use.

Next, the hemostatic property of apoV-HS was further evaluated in the heparinized rat femoral artery/vein bleeding model. The femoral artery and vein were exposed and cut by surgical scissors at 5 min post-heparin injection. After free bleeding for 10 s, apoV-HS was immediately put into the bleeding point (Fig. 6g). Importantly, the hemostatic ability of apoV-HS is mainly dependent on apoVs’ function rather than hydrogel sealing. The clotting time was significantly decreased in the apoV-HS group (2.5 ± 1 min) than in other groups (Fig. 6h). After complete hemostasis at 20 min, the total blood loss was calculated by weighting. The blood loss in the apoV-HS group was significantly lower than in other groups (Fig. 6i). All rats in the apoV-HS and Avitene™ group survived the surgery, while 25% of rats died in the no treated, PBS-HS, and Surgifoam® groups after surgery (Fig. 6j), but no statistically significant difference was observed for mortality. Furthermore, to verify the stability of apoV-HS stored at RT, we compared the procoagulant ability of apoV-HS from storage under RT for 2 months with − 80 °C for 2 months in rat femoral artery/vein bleeding model as above. There was no significant difference in the bleeding time and blood loss between these two groups (Additional file 1: Figure S4a, b), which confirmed that lyophilized apoV-HS were room-temperature stable for at least 2 months in vivo.

Normally, uncontrolled bleeding associated with coagulopathies occurs in a short time. The primary concern is to control blood loss quickly. Systemic delivery of EVs, such as intravenous injection, is often impractical. Moreover, several reports have verified that systemic infusion of TF^+^ or PS^+^ EVs leads to hypercoagulation, which results in thrombosis [60, 61]. Therefore, the topical application of apoV-HS is convenient and safe for fast hemorrhage control.

### ApoV-HS exhibits rapid hemostatic effect on heparinized rabbit femoral artery bleeding model

**Fig. 7:**
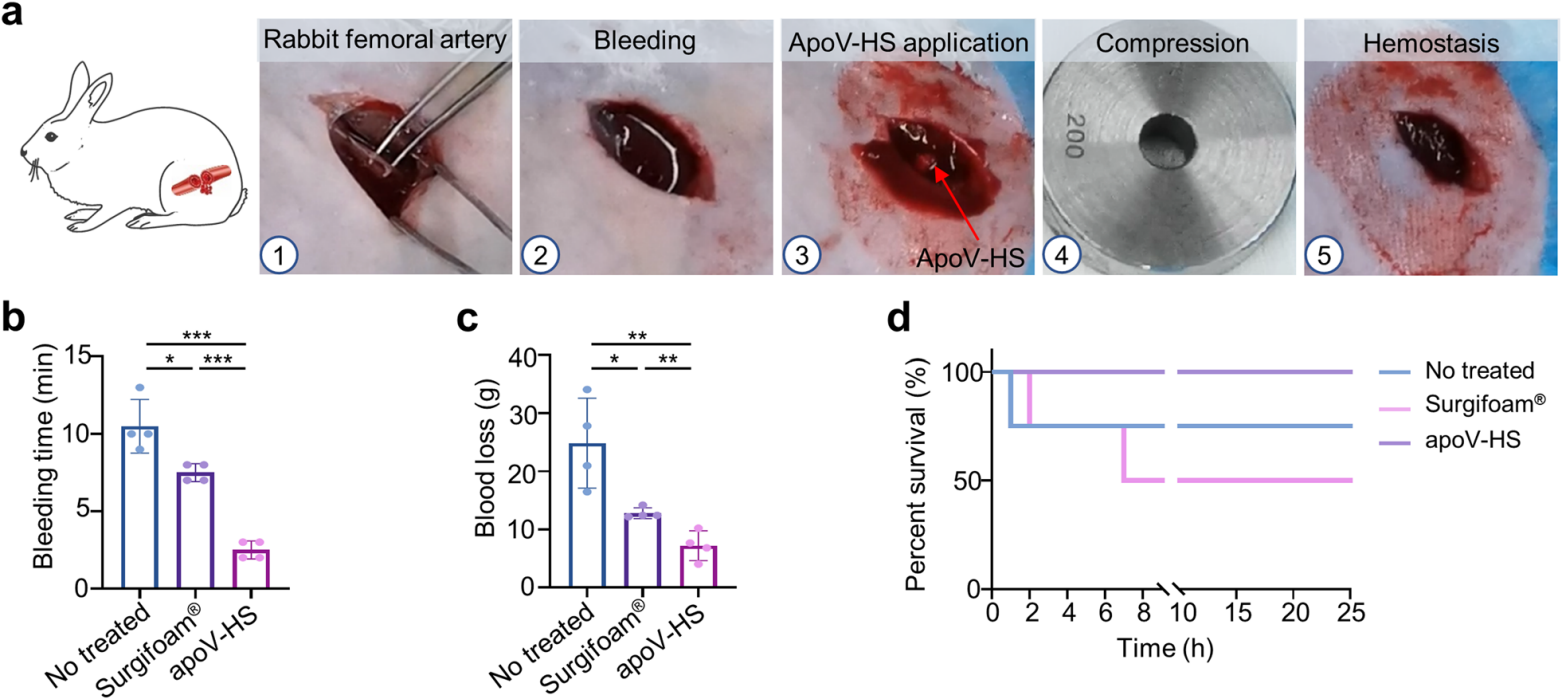
ApoV-HS exhibits rapid hemostatic effect on heparinized rabbit femoral artery bleeding model. **a** Digital photos showing hemostatic procedures treated with apoV-HS. **b**, **c** Bleeding time and blood loss of rabbit femoral artery bleeding models with indicated treatments. **d** The survival rate of rabbits. n = 4 per group. *p < 0.05; **p < 0.01; ***p < 0.001

To further evaluate the hemostatic ability of apoV-HS in vivo, we chose the heparinized rabbit femoral artery bleeding model, a large animal model, to simulate acute and uncontrolled bleeding. The rabbit femoral artery was exposed and punctured by a springe needle (1.2 mm in diameter) at 10 min post-heparin injection. After free bleeding for 10 s, the blood overflowing around the wound was quickly wiped away by medical gauzes for accurate assessment of blood loss, and hemostatic materials were rapidly placed on the bleeding point (Fig. 7a). Notably, in such a severe bleeding condition, this sponge absorbed enough blood and turned into a hydrogel, which could adhere tightly to the damaged femoral artery (Fig. 7a). Although some hydrogels have good adhesive capability, this property seems to fail in uncontrolled severe bleeding because they could not contact directly to the surface of tissues and organs due to the gushing blood [28, 62]. Notably, although some tissue adhesives (e.g., barnacle-inspired bioadhesive and citrate-based mussel-inspired bioadhesive) exhibited good adhesive property for hemostasis, the negative effects of high content of acrylic acid (tissue irritation) or dopamine (neurological effects) have limited their large-scale application and commercialization [63–65]. In contrast, apoV-HS could overcome these shortcomings because it could absorb blood and then turn into an adhesive hydrogel in a short time when injected into the injuries. Our experimental data showed that the hemostatic time (2.5 ± 0.5774 min) and blood loss amount (7.18 ± 2.557 g) in the apoV-HS group were significantly lower than those in the no treated (10.5 ± 1.732 min, 24.86 ± 7.749 g) and Surgifoam® groups (7.5 ± 0.5774 min, 12.79 ± 0.9319 g) (Fig. 7b, c). Rabbits treated with apoV-HS all survived the operation with no obvious adverse reaction, while 50% of rabbits treated by the Surgifoam® died of secondary bleeding after surgery (Fig. 7d). No statistically significant difference was observed for mortality. These results further imply that apoV-HS is a highly efficient hemostatic material in a large animal model.

Our previous study determined that the systematic infusion of MSC-apoVs could activate platelet function, trigger a shift from hypocoagulation to normal coagulation, and thus rescue the blood clotting disorder of haemophilia A [16]. The current study confirms that the topical application of MSC-apoVs, especially in the form of apoV-HS composite, can quickly staunch heavy bleeding in coagulopathic conditions. Based on these findings, apoV-based therapy is very promising for not only regulating physical coagulation state but also accelerating hemostasis to save lives.

Our current study has a few limitations which need to be addressed in future studies. First, larger animal models, such as porcine puncture wounds, are needed to further evaluate the hemostatic efficiency of apoV-HS, which will provide more accurate data for the clinical application on patients. Second, longer storage time at RT will need to verify the stability and validity of apoV-HS, which is beneficial for translating apoV-HS to a ready-to-use hemostat in the clinic. Third, for all of our studies, we have used only STS, which is a chemotherapy drug, to induce apoptosis and thus obtain apoVs. Future studies will be directed to explore other methods for inducing MSC apoptosis, such as mechanical forces, to acquire superior apoVs which is more suitable for clinical translation.

## Conclusion

In summary, this is the first study to illustrate apoVs’ superior procoagulant ability independent on hemocytes and cMPs, and fabricate a portable apoV-encapsulated adhesive hydrogel sponge for fast hemorrhage control in coagulopathies. With its high productivity, physiological stability, plasticity and injectability, excellent adhesivity, biocompatibility, and rapid coagulant property, apoV-HS provides a promising therapeutic product for heavy hemorrhage control in civilian and military populations. Furthermore, this is the first attempt to develop EV-based portable topical agents to rapidly cease severe bleeding at RT which also may be exploited for other biomedical applications.

## Experimental section

### Animals

Male Sprague–Dawley (SD) rats (7–8 weeks, 250–300 g) and male C57BL/6J mice (7–8 weeks) were obtained from the Experimental Animal Center of Sun Yat-sen University East Campus (Guangzhou, China), maintained in pathogen-free facilities. Male New Zealand rabbits (4–5 months, 2–2.5 kg) were obtained from Xinhua laboratory animal Center (Guangzhou, China), and housed in the conventional condition. All the animals were kept on standard 12 h light–dark cycles and received water and food *at libitum*. All experiments were in accordance with the ethics committees of Sun Yat-sen University (SYSU-IACUC-2022001534 and 2022002535). Animals were anesthetized by inhalation of isoflurane (1–2% isoflurane in oxygen, RWD, China) using a small animal anesthesia machine (RWD, China). Anesthesia was maintained by intramuscular administration of Zoletil®50 (50 mg kg^−1^, VIRBAC, France) and Xylazine Hydrochloride (50 mg kg^−1^, Sheng Da, China), according to the manufacturer’s instructions. Heparinized rat and rabbit models were achieved by intravenous heparin solution (Stemcell, Canada) administration (500 IU kg^−1^) [63].

### Antibodies and reagents

All antibodies, chemicals, and other reagents used in this study are listed in Additional file 1: Table S1.

### Isolation, culture, and characterization of UCMSCs

As in our previous study [66], human cords were obtained from full-term cesarean section surgery, with the informed consent of these donors. The cells used in this study were approved by the Medical Ethics Committee of the Hospital of Stomatology, Sun Yat-Sen University (KQEC-2021-59-01). After rinsed and removed vessels, the cords were cut into small pieces, followed by digestion with collagenase type I (2 mg ml^−1^, Worthington Biochemical, USA) and dispase II (4 mg ml^−1^, Roche Diagnostics, Germany) for 1 h at 37 °C. After that, the single-cell suspension was acquired by passing the cells through a 70 µm strainer (BD Biosciences, USA). All nucleated cells were seeded onto dishes (100 mm, Corning, USA) and cultured in alpha-Minimum Essential Medium (α-MEM, Invitrogen, USA) supplemented with 15% fetal bovine serum (FBS, Gibco, USA), 2 mM l-glutamine (Invitrogen, USA), and 1% penicillin/streptomycin (Invitrogen, USA) at 37 °C in 5% CO_2_. The medium of primary UCMSCs was replaced every 3 days. UCMSCs at the eighth-tenth passages were used for further experiments.

The UCMSCs were characterized by flow cytometric analysis. Briefly, UCMSCs were harvested and suspended in Stain buffer (BD Pharmingen™, USA) (5 × 10^5^ cells mL^−1^), incubated with PE-conjugated anti-human CD29 (1:100, BD Biosciences, USA), PE-conjugated anti-human CD44 (1:100, Biolegend, USA), PE-conjugated anti-human CD90 (1:100, BD Biosciences, USA), FITC-conjugated anti-human CD34 (1:100, BD Biosciences, USA), and PE-conjugated anti-human CD45 (1:100, BD Biosciences, USA) for 30 min at 4 °C. Data were analyzed by NovoExpress™ software (NovoCyte, USA).

### Induction of UCMSC apoptosis and isolation of apoVs

The UCMSCs with a confluence of 90–95% were washed twice with 1 X phosphate buffered saline (PBS, Servicebio, China), added with α-MEM containing different concentrations of staurosporine (STS, Enzo Life Sciences, USA) (250, 500, or 750 nM), and incubated at 37 °C for 12 h in 5% CO_2_, or added with α-MEM containing 500 nM STS and incubated for different time (4, 8, 12, or 16 h). ApoVs were isolated from the medium of apoptotic MSCs by sequential centrifugation (800*g* for 10 min, 2000*g* for 10 min, and 16,000*g* for 30 min) at 4 °C as we reported previously [67]. Finally, the pellet was washed once with 0.22 μm-filtered PBS to purify apoVs.

### Isolation of Exos

As in our previous report [16], UCMSCs were washed twice with PBS and then cultured in α-MEM for 48 h at 37 °C. Exos in the culture supernatants were isolated by sequential centrifugation (800*g* for 10 min, 2000*g* for 10 min, 16,000*g* for 30 min, and 120,000*g* for 120 min) at 4 °C.

### Identification of UCMSC-derived apoVs

#### Cryo-electron microscopy (Cryo-EM)

The morphology of apoVs was observed by Cryo-EM (Thermo Fisher, USA) as we previously described [16, 68]. In brief, after sample preparation, images of apoVs were collected at a dose rate of 40 e/pixel/s, exposed for 1 s. The pixel size at the object scale was 1.584 Å (nominal magnification 92 K) and 2.557 Å, with the defocus set at about -3 μm.

#### Nanoparticle tracking analysis (NTA)

NTA was performed by ZetaView® PMX120 (Particle Metrix, Germany) to analyze the total numbers, size distribution, and membrane potentials of apoVs and Exos. ApoVs or Exos were diluted by ultrapure water and measured at 11 different positions with a medium video quality setting. The data was calculated by ZetaView® analysis software.

#### Flow cytometric analysis

The surface markers of apoVs were identified as in our previous research [16]. In brief, apoVs were harvested and suspended in Stain buffer and incubated with PE-conjugated anti-human TF (1:50, Biolegend, USA), PE-conjugated anti-human Fas (1:50, Biolegend, USA), PE-conjugated anti-human CD9 (1:50, Biolegend, USA), PE-conjugated anti-human CD63 (1:50, Biolegend, USA), and PE-conjugated anti-human CD81 (1:50, Biolegend, USA), PE-conjugated anti-human CD29 (1:50), PE-conjugated anti-human CD44 (1:50), PE-conjugated anti-human CD90 (1:50), FITC-conjugated anti-human CD34 (1:50), and PE-conjugated anti-human CD45 (1:50) for 30 min at 4 °C. For PS detection, apoVs were suspended in Annexin V Binding Buffer (BD Pharmingen™, USA) and stained with PE-Annexin V (1:50) at 4 °C for 20 min. The positive rate of apoVs were analyzed by NovoExpress™ software.

#### Western blotting

Cells, apoVs, and Exos were lysed by the RIPA Lysis Buffer System (Santa Cruz Biotechnology, USA) to extract protein. After quantification of protein concentration through the Pierce™ BCA Protein Assay Kit (Thermo Scientific, USA), identical amounts of protein samples were loaded onto the NuPAGE™ 4%–12% Mini Protein Gel (Invitrogen, USA) and transferred to polyvinylidene fluoride membranes (Millipore, USA). The membranes were blocked by 5% bovine serum albumin (BSA, Sigma-Aldrich, USA) for 1 h at RT and incubated with primary antibodies (1:200–1000 dilution) overnight at 4 °C. After washing with TBS containing 0.1% Tween®20 (Amresco, USA) for 3 times, the membranes were incubated with species-related HRP-conjugated secondary antibodies (1:10,000 dilution) for 1 h at RT. The bands were visualized through SuperSignal™ West Pico PLUS Chemiluminescent Substrate kit (Thermo Scientific, USA) or SuperSignal™ West Femto Maximum Sensitivity Substrate kit (Thermo Scientific, USA), and then evaluated by a ChemiDoc™ MP imaging system (BIO-RAD, USA).

### Blood-related component collection and preparation

Rat circulating whole blood (CWB) was collected from the inferior vena cava of healthy male rats using anticoagulant tubes (Kangvis, China) (3.8% sodium citrate: blood = 1: 9). The CWB was centrifuged (3000 rpm for 15 min at RT) to acquire Rat packed red blood cells (RBC). 5% hematocrit of RBC suspension was prepared by diluting packed RBC (100 μL) with PBS (2 mL). Rat normal plasma (NP) was obtained from rat CWB by centrifugation (800*g* for 10 min at RT). Rat platelet-poor plasma (PPP) was obtained from NP after centrifugation (3000*g* for 10 min at 4 °C). Rat microparticle-depleted plasma (MDP) was obtained by filtering PPP (0.1 μm) as reported previously [40]. Human MDP was acquired from the blood of healthy individuals using the same procedure as above and the donors were informed consent.

### Blood and plasma clotting experiment

The blood and plasma clotting experiment was performed according to a previous report with some modifications [26]. Briefly, blood or plasma (40 μL) was incubated with apoVs (12.5 μL) in the flow tube for 3 min at 37 °C. After that, CaCl_2_ (0.2 M, 7 μL, Sigma-Aldrich, USA) was added and clotting time was recorded (within 1200 s), with PBS or Exos as control.

### TF and PS blocking

The purified apoVs were resuspended in Annexin V binding buffer (2.4 × 10^8^ particles, 12.5 μL), incubated with Annexin V (1 µg mL^−1^, AV, Sino biological, China) or Tissue factor pathway inhibitor (32 µg mL^−1^, TFPI, Sino biological, China) for 30 min at 4 °C, then used for the plasma clotting assays.

### Transfection of siRNA in UCMSCs

To abrogate TF expression in UCMSC-derived apoVs, the UCMSCs (50–70% confluent) were transfected with siRNA-negative control (si-NC) or siRNA-TF (si-TF) (Santa Cruz Biotechnology, USA) using the Lipofectamine™ RNAiMAX Transfection Reagent (Invitrogen, USA) and Opti-MEM Medium (Gibco, USA), following the manufacturer’s directions. Transfection efficiency was evaluated at 48 h post-transfection via western blotting. UCMSCs transfected with siRNA (120 nM) for 72 h were used for apoV isolation.

### Lyophilization of apoVs

According to previous research [17], apoVs (1.2 × 10^10^ particles, 100 μL) were added to lyoprotectants (400 μL), containing 100 mM d-(+)-Trehalose dihydrate (Trehalose, Macklin, China) and 5% polyvinylpyrrolidone 40 (PVP 40, Coolaber, China). After being frozen overnight at − 80 °C, the mixture above was lyophilized using a − 80 °C freeze drier (Shengwei, China) for 30 h to obtain lyophilized apoVs, which were stored in sealed boxes to be moisture-proof at − 80 °C or RT. Before use, lyophilized apoVs in each tube were rehydrated by adding ultrapure water (500 μL).

### Synthesis of apoV-encapsulated hydrogel sponge (apoV-HS)

#### Manufacture of phenylboronic acid grafted oxidized hyaluronic acid (PBA-HA)

Based on previous reports [32, 56], hyaluronic acid sodium salt (1 g, HA, Yuanye, China) was dissolved in deionized water (DI, 100 mL) under stirring (200 rpm), and then 3-Aminobenzeneboronic acid (3-APBA, Macklin, China) and 4-(4,6-Dimethoxy-1,3,5-triazin-2-yl)-4-methyl morpholinium chloride (DMTMM, Macklin, China) were added, respectively. The molar ratio of 3-APBA, DMTMM, and HA was 1:1:1. The mixture was stirred (200 rpm for 24 h at RT), and then dialyzed (MWCO 3500 Da) using DI for 3 days to eliminate the unreacted substance. The dialyzed solution was lyophilized to obtain white spongy solid phenylboronic acid grafted hyaluronic acid using a − 50 °C freeze drier (LABCONCO, USA). Phenylboronic acid grafted hyaluronic acid (1 g) was dissolved in DI (100 mL) followed by sodium periodate (0.25 M, 10 mL, Macklin, China) added dropwise. After reacting in dark for 24 h at RT, glycol (1 mL, Macklin, China) was added to terminate the reaction, and then transferred into the dialysis membrane (MWCO 3500 Da) to purify PBA-HA. After 3 days of dialysis using DI, the purified PBA-HA was finally lyophilized by a − 50 °C freeze drier and stored at 4 °C.

#### Synthesis of apoV-HS

PBA-HA (60 mg) was dissolved in PBS (500 μL) at RT for 1 h, and poly(vinyl alcohol) (40 mg, PVA, Aladdin, China) was dissolved in PBS (500 μL) at 95 °C for 1 h to prepare 12% PBA-HA and 8% PVA, respectively. ApoVs (4 × 10^10^ particles, 50 μL) or PBS (50 μL) was added into lyoprotectants (200 μL), containing 200 mM trehalose and 10% PVP40, with supplements of 12% PBA-HA (125 μL) and 8% PVA (125 μL) to formulate apoVs hydrogel or PBS hydrogel. The hydrogels were frozen overnight at − 80 °C and then lyophilized for 30 h using a − 80 °C freeze drier to obtain apoV-HS or PBS hydrogel sponge (PBS-HS).

### Water absorption and adhesive test

As previous studies reported [26], apoV-HS (60 mg) was compressed and injected into DI (3 mL) to formulate hydrated apoV hydrogel. The hydrogel was weighted at set time points. All samples were replicated (n = 3) and the swelling ratio was calculated according to following formula:

$${\text{Swelling ratio}}\left( \% \right) = ({\text{Ms}} - {\text{Md}})/{\text{Md}} \times {1}00\%$$

Ms and Md represented the weight of sample in swollen and dry states, respectively.

The apoV hydrogel was put on a slide (CITOTEST, China). Afterwards, the heart, liver, spleen, lung, kidney, or skin (6 mm × 6 mm) of a mouse were placed on the hydrated apoV hydrogel, respectively. The slide was then inverted to observe the adhesive capability of the hydrated apoV hydrogel.

### Blood clot index (BCI)

BCI was calculated according to a previous report with some modifications [26]. Briefly, rat CWB (30 μL) and CaCl_2_ (0.2 mol L^−1^, 7 μL) was slowly dropped on the surface of the comparable volume samples or plate (negative control), respectively, and incubated at 37 °C for 5 min. PBS (5 mL) was carefully added without perturbing the blood clot. After that, specimens were shaken gently (120 rpm for 5 min at 37 °C). The absorption of the solution at 540 nm was recorded by Multimode Microplate Reader (BioTek SynergyH1, USA). Surgifoam® and Avitene™ were selected as the positive control.

The BCI of different materials was measured by the following formula:

$${\text{BCI}}\left( \% \right) = \left( {{\text{OD}}_{{{\text{sample}}}} /{\text{OD}}_{{\text{reference value}}} } \right) \times {1}00\%$$

where OD_sample_ was the absorbance of the solution from the samples at 540 nm, and OD_reference value_ was the absorbance of the solution from the negative control at 540 nm.

### ApoV release profile of apoV-HS in vitro

As previously reported [22], apoVs, labeled by PKH26 Red Fluorescent Cell Linker kit (Sigma-Aldrich, USA) following the manufacturers’ directions, were used to synthesize PKH26-labeled apoV-HS After being immersed in PBS (1 mL) at 37 °C, the supernatant of PKH26-labeled apoV-HS was collected in predetermined time points (1 min, 3 min, 5 min, 7 min, 10 min, 1 h, 3 h, 7 h, 10 h, and 24 h). The fluorescence intensity of the supernatant was measured at 570 nm by Multimode Microplate Reader.

### Scanning electron microscope

ApoV-HS and PBS-HS were compressed and injected into DI (1 mL) in an Eppendorf tube. After 10 min, ApoV-HS and PBS-HS were hydrated, frozen at − 80 °C overnight, and then subjected to lyophilization for 30 h using a − 80 °C freeze drier. The freeze-dried PBS-HS and apoV-HS were sprayed with gold by Sputter Coater (MC1000, Hitachi, Japan) for electric conduction, and observed by Scanning electron microscope (SEM, Quanta200, Thermo Fisher, USA).

### Biocompatibility evaluation in vitro

PBS-HS or apoV-HS (60 mg) were incubated in High glucose Dulbecco’s Modified Eagle Medium (25 mL, DMEM, Biosharp, China) supplemented with 10% FBS and 1% penicillin–streptomycin at 37 °C for 24 h to prepare the extracts, as in previous report [63]. Cell viability was measured by contacting extract.

#### CCK-8 assay

The cell counting kit-8 (CCK-8, Dojindo, Japan) was used to assess cell proliferation according to the manufacturer’s protocol. Specifically, L929 cells (mouse fibroblasts) were seeded (1 × 10^4^ per well) into 96-well plates (Corning, USA) and treated with the extracts or normal culture medium for 24 h, 48 h, or 72 h, respectively. CCK-8 (10 μL) was added to each well and incubated for 2 h. The absorbance of the CCK-8 solution was detected at 450 nm by Multimode Microplate Reader.

#### EdU proliferation assay

The cell proliferation was further performed by the kFluor488-EdU Cell Proliferation Detection Kit (KeyGEN, China). Briefly, L929 cells (5 × 10^4^ per well) were seeded into 24-well plates (Corning, USA), cultured with the extracts or normal culture medium for 24 h, and subsequently incubated with EdU for 2 h. Cells were fixed by 4% paraformaldehyde (PFA, Sigma-Aldrich, USA), then underwent staining according to the manufacturer’s instructions, and mounted with DAPI (Abcam, USA). The cells were imaged using fluorescence microscopy (ZEISS, Axio Observer 5, Germany), and the number of proliferating cells was calculated.

### In vivo experiments

The PBS-HS and apoV-HS used in all animal experiments were prepared by aseptic techniques [63].

#### In vivo biocompatibility evaluation

The rat subcutaneous implantation experiment (n = 6) was according to a previous study [63]. After anesthesia and hair removal of the back, a subcutaneous pocket was generated by dorsal skin incisions (1 cm in length) and blunt dissection, and PBS-HS (60 mg) or apoV-HS (60 mg) was implanted into it, with no implantation as control. Three implants were placed in each rat, ensuring no overlap between the subcutaneous pockets. Next, the wounds were closed by interrupted sutures (4–0, JZ, China) and saline (5 mL) was injected subcutaneously. After 2 weeks and 4 weeks, the rats were euthanized by cervical dislocation under anesthesia to collect the implants and adjacent tissue. There were still residues that could be visually distinguished. Next, the samples were fixed by 4% PFA for hematoxylin–eosin (HE) staining and observed by microscope (ZEISS, Axio Observer 5, Germany). Infiltrating inflammatory cells were detected by IF staining. In brief, the sections were blocked with 5% BSA for 1 h at RT, then incubated with primary antibody CD3 (1:100) overnight at 4 °C. Next, the sections were washed and stained with Alexa Fluor 561-conjugated goat anti-rabbit secondary antibody (1:200) for 1 h at RT, then washed and counterstained with DAPI.

#### Heparinized rat liver bleeding model

As previously reported [55], after injection of heparin solution intravenously for 30 min, median laparotomy was performed in rats, and the left anterior liver lobe was exposed. Interstitial fluid surrounding the liver was wiped off with medical gauzes (Zeguan, China) to ensure the accuracy of blood loss. The biopsy punch (Kai, Japan) was used to create a cylindrical wound (8 mm in diameter, 5 mm in depth) in the left anterior lobe of the liver. The surrounding blood was wiped away to ensure accurate measurement of blood loss, and apoV-HS was quickly applied on the defect site. For comparison, a comparable volume of PBS-HS, commercial hemostats (Avitene™ and Surgifoam®), and no treated were selected as control. Each group contained 4 animals. The weighed filter paper was placed under the liver to absorb blood. The bleeding time was recorded (within 5 min), and the filter paper was weighed again to calculate blood loss. After 4 weeks, all explants and adjacent hepatic tissue were extracted, fixed by 4% PFA and embedded in paraffin. Paraffin sections (4 μm) were prepared for HE staining and observed by microscope. IF staining was used to show CD3^+^ T cells in the liver.

#### Heparinized rat femoral artery/vein bleeding model

As described previously [69], the left femoral artery and vein of the rats were exposed and cut at 5 min post-heparin solution injection. After 10 s free bleeding, blood around the bleeding point was wiped away, and apoV-HS (60 mg), PBS-HS (60 mg) or the comparable volume of commercial hemostats (Avitene™ and Surgifoam®) were applied on the bleeding vessel immediately. Meanwhile, pre-weighed gauzes and a weight (200 g) was placed on the wound as constant pressure. The bleeding time (within 20 min) and blood loss were recorded and each group contained four rats.

#### Heparinized rabbit femoral artery bleeding model

According to a previous study [55], the left femoral artery of rabbits was exposed at 10 min post-heparin injection and surrounding interstitial fluid was removed by medical gauzes. Severe arterial hemorrhage was executed by puncturing the femoral artery with a syringe needle (1.2 mm in diameter), with free bleeding for 10 s. Blood around the wound was quickly wiped off to ensure an accurate assessment of blood loss. ApoV-HS (120 mg) was applied to the bleeding artery immediately and covered with pre-weighed gauzes and a weight (200 g). The hemorrhage was checked every minute. No treated and a comparable volume of Surgifoam® were selected as control groups, and each group contained 4 rabbits.

### Statistical analysis

Statistical and graph analyses were performed using GraphPad Prism 9.0 (IBM, USA). For two-group comparisons, significance was assessed by unpaired Student’s* t* test. Differences among multiple groups were analyzed by one-way ANOVA with Tukey’s post hoc test. Survival rate was analyzed using log-rank Mantel-Cox test for two and multiple group comparisons. All the data were presented as mean ± standard deviation (SD). ns, no significant. *p < 0.05; **p < 0.01; ***p < 0.001.

### Supplementary Information

**Additional file 1: Figure S1.** Flow cytometric analysis of the surface markers of human umbilical cord mesenchymal stem cells (UCMSCs). **Figure S2.** Characteristics of apoptotic vesicles (apoVs) derived from UCMSCs. **Figure S3.** The expression of tissue factor (TF) in UCMSCs and apoVs. **Figure S4.** Hemostatic effect of lyophilized apoV-encapsulated hydrogel sponge (apoV-HS) in heparinized rat femoral artery/vein bleeding model. **Table S1.** Reagents and Resources Table.

## Data Availability

All data and materials are showed in the paper and further inquiries can be directed to the corresponding author.
