# Supplementary material for: Lyophilized apoptotic vesicle-encapsulated adhesive hydrogel sponge as a rapid hemostat for traumatic hemorrhage in coagulopathy
Source: J Nanobiotechnology. 2023 Nov 3;21:407. doi: 10.1186/s12951-023-02128-2 (PMC10623807; doi:10.1186/s12951-023-02128-2)
Supplement: Supplementary file 1 — Additional file 1: Figure S1. Flow cytometric analysis of the surface markers of human umbilical cord mesenchymal stem cells (UCMSCs). Figure S2. Characteristics of apoptotic vesicles (apoVs) derived from UCMSCs. Figure S3. The expression of tissue factor (TF) in UCMSCs and apoVs. Figure S4. Hemostatic effect of lyophilized apoV-encapsulated hydrogel sponge (apoV-HS) in heparinized rat femoral artery/vein bleeding model. Table S1. Reagents and Resources Table. [file 12951_2023_2128_MOESM1_ESM.docx]

Additional information

**Lyophilized apoptotic vesicle-encapsulated adhesive hydrogel sponge as a rapid hemostat for traumatic hemorrhage in coagulopathy**

Yexiang Jiang^1, #^, Meng Hao^1, #^, Fenglin Jiang^2^, Jiwu Li^3^, Kunkun Yang^4^, Can Li^1^, Lan Ma^1^, Shiyu Liu^5^, Xiaoxing Kou^1^, Songtao Shi^1,^ *, Xin Ding^2,^ *, Xiao Zhang^4,^ *, Jianxia Tang^3,^ *

^1^Hospital of Stomatology, Guanghua School of Stomatology, Sun Yat-sen University, South China Center of Craniofacial Stem Cell Research, Guangdong Provincial Key Laboratory of Stomatology, Guangzhou, 510055, China.

^2^School of Pharmaceutical Science (Shenzhen), Shenzhen Campus of Sun Yat-sen University, Shenzhen, 518107, China.

^3^Hunan Key Laboratory of Oral Health Research & Hunan Clinical Research Center of Oral Major Diseases and Oral Health, Xiangya School of Stomatology, Xiangya Stomatological Hospital, Central South University, Changsha, 410000, China.

^4^Department of Prosthodontics, Peking University School and Hospital of Stomatology, National Center of Stomatology, National Clinical Research Center for Oral Diseases, National Engineering Laboratory for Digital and Material Technology of Stomatology, NHC Key Laboratory of Digital Technology of Stomatology, Beijing Key Laboratory of Digital Stomatology, Beijing, 100081, China.

^5^State Key Laboratory of Military Stomatology & National Clinical Research Center for Oral Diseases & Shaanxi International Joint Research Center for Oral Diseases, Center for Tissue Engineering, School of Stomatology, The Fourth Military Medical University, Xi’an, PR China.

^#^ These authors contributed equally to this work.

*** Corresponding authors:**

**Songtao Shi**, E-mail: shisongtao@mail.sysu.edu.cn.

**Xin Ding**, E-mail: dingxin3@mail.sysu.edu.cn.

**Xiao Zhang**, E-mail: kqxiaozhang@hsc.pku.edu.cn.

**Jianxia Tang**, E-mail: jianxiatang@csu.edu.cn.

**Additional figures and legends**

**
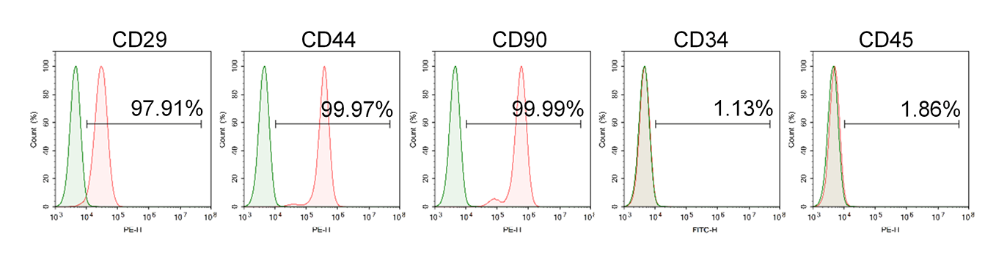
**

**Figure S1.** **Flow cytometric analysis of the surface markers of human umbilical cord mesenchymal stem cells (UCMSCs).**

**
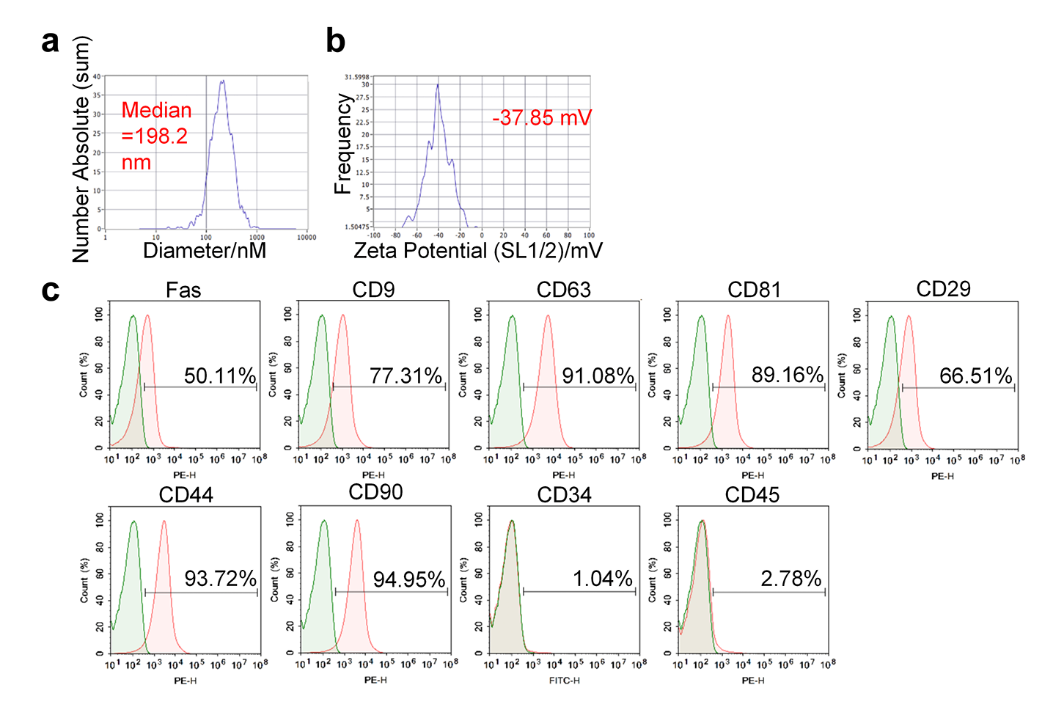
**

**Figure S2.** **Characteristics of apoptotic vesicles (apoVs) derived from UCMSCs.**

**a, b)** NTA exhibiting the size distribution **(a)** and membrane potential **(b)** of apoVs. ApoVs were collected from UCMSCs treated with STS (500 nM) for 12 h. **c)** Flow cytometric analysis indicating surface marker expression of apoVs.

**
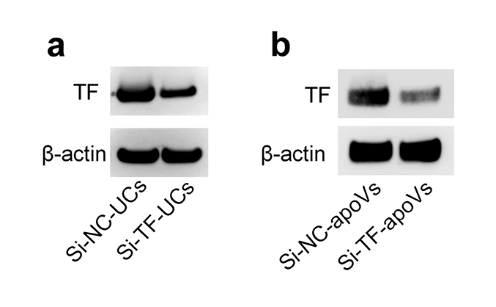
**

**Figure S3.** **The expression of tissue factor (TF) in UCMSCs and apoVs.**

**a)** si-NC-UCs, UCMSCs treated by siRNA-NC; si-TF-UCs, UCMSCs treated by siRNA-TF. **b)** si-NC-apoVs, apoVs derived from UCMSCs treated by siRNA-NC; si-TF-apoVs, apoVs derived from UCMSCs treated by siRNA-TF.


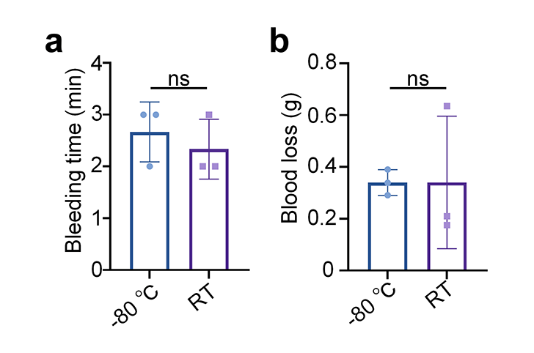


**Figure S4.** **Hemostatic effect of lyophilized apoV-encapsulated hydrogel sponge (apoV-HS) in heparinized rat femoral artery/vein bleeding model.**

**a, b)** Bleeding time **(a)** and blood loss **(b)** for rat femoral artery/vein bleeding models treated with apoV-HS. -80 °C, apoV-HS from storage under -80 °C for 2 months; RT, apoV-HS from storage under room temperature for 2 months. ns, no significant.

**Table S1. Reagents and Resources Table.**

| **Reagents or Resources** | **Source** | **Category Number** |
| --- | --- | --- |
| PE anti-human TF | Biolegend | 365204 |
| PE anti-human CD44 | Biolegend | 103023 |
| PE anti-human CD9 | Biolegend | 312105 |
| PE anti-human CD63 | Biolegend | 353003 |
| PE anti-human CD81 | Biolegend | 349505 |
| PE anti-human Fas | Biolegend | 305607 |
| PE anti-human CD29 | BD Biosciences | 557332 |
| PE anti-human CD90 | BD Biosciences | 561970 |
| FITC anti-human CD34 | BD Biosciences | 555821 |
| PE anti-human CD45 | BD Biosciences | 555483 |
| β-actin | Sigma-Aldrich | A1978 |
| CD44 | Abcam | ab189524 |
| FAS | Abcam | ab82419 |
| Integrin α5 | Abcam | ab150361 |
| CD9 | Abcam | ab236630 |
| Lamin B1 | Abcam | ab133741 |
| Syntenin-1 | Abcam | ab19903 |
| Calnexin | Santa Cruz Biotechnology | sc-23954 |
| TF | Santa Cruz Biotechnology | sc-374441 |
| Calreticulin | Cell Signaling Technology | 12238S |
| RPS25 | Proteintech | 23599-1-AP |
| Mouse anti-rabbit IgG-HRP-linked Antibody | Santa Cruz Biotechnology | sc-2357 |
| Anti-rat IgG HRP-linked Antibody | Cell Signaling Technology | 7077S |
| Staurosporine | Enzo Life Sciences | ALX-380-014 |
| Triton^TM^ X-100 | Sigma-Aldrich | X100-100ML |
| L -glutamine | Invitrogen | 35050-061 |
| Penicillin-Streptomycin | Invitrogen | 15140-122 |
| Tryple^TM^ Express Enzyme | Invitrogen | 12605-010 |
| Stain buffer | BD Pharmingen™ | 554657 |
| 10 X Annexin V Binding Buffer | BD Pharmingen™ | 556454 |
| NuPAGE MES SDS Running Buffer | Invitrogen | NP0002 |
| NuPAGE Transfer Buffer | Invitrogen | NP00061 |
| PageRuler™ Prestained Protein Ladder | Invitrogen | 26616 |
| Novex™ Sharp Pre-stained Protein Standard | Invitrogen | LC5800 |
| Bovine serum albumin | Sigma-Aldrich | B2064 |
| TF siRNA (h) | Santa Cruz Biotechnology | sc-44984 |
| Lipofectamine™ RNAiMAX Transfection Reagent | Invitrogen | 13778 |
| Polyvinylpyrrolidone | Coolaber | CP9251 |
| D-(+)-Trehalose dihydrate | Macklin | D-(+)-Trehalose dihydrate |
| Hyaluronic acid sodium salt | Yuanye | S28512 |
| Poly(vinyl alcohol) | Aladdin | P105126 |
| 3-Aminobenzeneboronic acid | Macklin | A823222 |
| 4-(4,6-Dimethoxy-1,3,5-triazin-2-yl)-4-methyl morpholinium chloride | Macklin | D807380 |
| Annexin V | Sino Biological | 10448-HNAE |
| Human TFPI Protein | Sino Biological | 10564-H08H |
| PE Annexin V | Biolegend | 640908 |
| Collagenase type I | Worthington Biochemical | LS004197 |
| Dispase II | Roche Diagnostics | 4942078001-1 |
| Mounting medium with DAPI | Abcam | ab104139 |
| NuPAGE™ LDS Sample Buffer | Invitrogen | NP0007 |
| Factor II-deficient plasma | Prolytix | FII-ID-50 |
| Factor V-deficient plasma | Prolytix | FV-ID-50 |
| Factor X-deficient plasma | Prolytix | FX-ID-50 |
| Factor VII-deficient plasma | Prolytix | FVII-ID-1 |
| Factor VIII-deficient plasma | Prolytix | FVIII-CD-500 |
| Factor IX-deficient plasma | Prolytix | FIX-ID-1 |
| Factor XI-deficient plasma | Prolytix | FXI-ID-50 |
| Factor XII-deficient plasma | Prolytix | FXII-ID-1 |
| Isoflurane | RWD | R510-22-70 |
| Zoletil^®^50 | VIRBAC | 50 |
| Xylazine Hydrochloride | Sheng Da | 2365 |
| Heparin solution | Stemcell | 07980 |
| 10 X Phosphate Buffered Saline | Servicebio | G4207 |
| CaCl2 | Sigma-Aldrich | C5670 |
| Paraformaldehyde | Sigma-Aldrich | 441244 |
| Glycol | Macklin | E808737 |
| Sodium periodate | Macklin | S817518 |
| Tween^®^20 | Amresco | 0777 |
| RIPA Lysis Buffer System | Santa Cruz Biotechnology | sc-24948 |
| NuPAGE^TM^ 4 to 12%, Mini Protein Gel, 12-well | Invitrogen | NP0322BOX |
| Pierce^TM^ BCA Protein Assay Kit | Thermo Scientific | 23225 |
| SuperSignal^TM^ West Femto Maximum Sensitivity Substrate | Thermo Scientific | 34095 |
| SuperSignal^TM^ West Pico PLUS Chemiluminescent Substrate | Thermo Scientific | 34580 |
| PKH26 Red Fluorescent Cell Linker kit | Sigma-Aldrich | PKH26PCL |
| Cell Counting Kit-8 | DOJINDO | ck04 |
| kFluor488-EdU Cell Proliferation Detection Kit | KeyGEN | KGA331-1000 |
| Alpha-Minimum Essential Medium | Invitrogen | 12571-048 |
| Fetal bovine serum | Gibco | 10099141 |
| Opti-MEM Medium | Gibco | 31985070 |
| High Glucose Dulbecco’s Modified Eagle Medium | Biosharp | BL304A |
